# Narcissistic traits in young people and how experiencing shame relates to current attachment challenges

**DOI:** 10.1186/s12888-021-03249-4

**Published:** 2021-05-11

**Authors:** Charlotte C. van Schie, Heidi L. Jarman, Samantha Reis, Brin F. S. Grenyer

**Affiliations:** 1 Illawarra Health and Medical Research Institute and the School of Psychology, University of Wollongong, Northfields Avenue, Wollongong, NSW 2522 Australia; 2 The Reflective Space: Clinical Psychology & Psychotherapy Services, PO Box 778 Milsons Point, Sydney, NSW 1565 Australia

**Keywords:** Vulnerable narcissism, Grandiose narcissism, Shame, Attachment, Ideal self, Self-compassion, Young people, Mediation analysis

## Abstract

**Background:**

Young people with pathological narcissistic traits may have more maladaptive ways of relating to themselves and others. In this study, we investigated how the experience of shame may be a mechanism by which vulnerable and grandiose pathological narcissism relates to negative and positive internalised models of the self and others, manifested as attachment styles.

**Methods:**

Participants (*N* = 348) were young people who reported on pathological narcissism, the experience of shame and their model of self and others (secure, dismissive, preoccupied and fearful attachment). Mediation of the experience of shame between vulnerable and grandiose narcissism on the one hand and secure, dismissive, preoccupied and fearful attachment on the other hand, was tested using a path model.

**Results:**

Shame mediated the relationship between vulnerable narcissism and a more negative model of others and self (i.e. less secure, more fearful and more preoccupied in attachment). Higher grandiose narcissism traits were related to a more positive model of others and self (i.e. more secure attachment) and were unrelated to the experience of shame.

**Conclusions:**

Young people with vulnerable narcissism traits tended to report more shame, and struggled to be close to others. It may be that shame experiences highlight a discrepancy between the ideal and actual self that may contribute to a more insecure attachment style. A good working alliance and fostering self-compassion may counter some negative effects of shame in those most vulnerable, but dismissal in those most grandiose presents a clinical conundrum requiring further research.

**Supplementary Information:**

The online version contains supplementary material available at 10.1186/s12888-021-03249-4.

## Introduction

Global estimates are that personality disorder affects around 7.8% of the population [1] and poses a high burden to both the person affected and to people around them [2, 3]. Psychological treatment for borderline personality disorder shows some efficacy [4], yet there are few clinical trials of treatment for narcissistic personality disorder (NPD) [5]. Research on the clinical implications of pathological narcissism are in their infancy despite the long history of the disorder [6]. There is an urgent need to further understand where pathological narcissism may set up a cascade of self and interpersonal difficulties that may guide future therapies, particularly in youth [7, 8].

Pathological narcissism is understood to have a core of entitlement and self-importance and has vulnerable traits such as contingent self-esteem and grandiose traits such as exploitativeness [9–11]. Certain narcissism traits may be adaptive, even important, for going through the transition phase of adolescence and emerging adulthood [12]. There appears to be a normative increase in narcissism traits in late adolescence [13, 14]. Feelings of omnipotence may then be conducive to personal growth in the process of separating from parents and the individuation of self [12, 15]. However, it may also be particularly in this phase of life that narcissism traits become pathological and in a more extreme form may develop into NPD [16–18]. Pathological narcissism may be observed as young people struggling to build genuine relationships and rigidly holding on to an extreme ideal self [16]. In normal development, the ideal self matches the young person’s talents and characteristics, is flexible and amenable to change, and may provide a sense of agency [16]. An ideal grandiose self, however, may instead act as a constant threat that one cannot measure up to [16].

These positive yet fragile selves may have their origin in childhood with overprotective and overvaluing parenting relating to more grandiose and vulnerable narcissism traits [19, 20]. This parenting environment may lead to young people being dependent on others for feedback and guidance in order to sustain a positive self-view [21–24]. At the same time, people higher in narcissistic traits may be quite reactive to social interactions [25]. In seeking affirmation from others of their positive yet fragile selves [9, 26–29], people high in narcissism may impair the very relationships they need by prioritising self-affirmation over being liked [6, 23]. It is important to understand how these difficulties in self and other functioning in young people with narcissism traits come about to inform treatment and prevention.

One reason that people high in narcissism traits react strongly to interpersonal interactions is that these may reveal a gap between the ideal self and the actual self. This discrepancy becomes more painful as the gap between the actual and ideal self becomes larger e.g. when the ideal self is grandiose [30]. Feelings of shame may highlight this discrepancy between the ideal and actual self [16, 30, 31]. Shame is seen as a central aspect of pathological narcissism that has been implicated in its development, functioning and treatment [26, 30, 32, 33] and is a common experience among people with NPD [34]. Shame is a self-conscious emotion that brings awareness to undesirable aspects of the self that are seen as stable over time and global across situations [35–38]. This is different from other self-conscious emotions such as guilt which is tied to a specific negative behaviour and does not encompass the whole self [39]. Moreover, shame can disrupt the functioning of the self whereby a grandiose self and more negative characteristics are not well integrated resulting in a more rigid and fragile unrealistic self [30].

At the same time, shame is an interpersonal emotion that can disrupt social interactions [36, 37, 40]. Where guilt may motivate behaviour to repair relationships, shame seems to motivate withdrawal from others [39, 41]. Although, shame may also motivate mending behaviour [42, 43], it is when people have difficulty with self-regulation or are in a situation where the self is threatened, that shame may lead to withdrawal or even aggressive behaviour [36, 42, 44, 45]. For example, one study showed that children higher in narcissism responded with more aggression after their failure in performing was shamed [46]. The experience of shame may thus be a candidate mechanism by which young people with higher narcissism traits may experience disturbances in the self and difficulties in relating to others.

Theories suggest that shame is a core affect of vulnerable narcissism [47]. Research supports a relation between vulnerable narcissism and the experience of shame [29, 48–50]. Shame may be experienced when one receives negative feedback that feels undeserved [50]. Receiving positive feedback may also relate to experiencing shame when it does not match with the individuals own perception [50, 51]. Interestingly, this may support the notion that it is not so much the valence of feedback (i.e. negative or positive) that elicits shame but whether a discrepancy between and ideal and actual self is experienced. Facets of vulnerable narcissism that have previously been related to shame are relying on others for feedback (i.e., contingent self-esteem) and devaluing others when they do not provide recognition, whereas entitlement rage may be related to experiencing less shame [49]. It has been proposed that grandiose narcissism may be related to the denial of shame [47] with some studies finding a negative relationship between shame and grandiose narcissism [35, 52] and higher levels of implicit shame in people with NPD compared to people with BPD [34]. However, other studies have not found an association with shame [48] or have found increased experiences of shame specifically in relation to grandiose fantasies [29, 49] i.e., feeling shame for having grandiose needs and ambitions [47]. Thus, research on the association between grandiose narcissism and shame is far from conclusive.

In the context of narcissism research, attachment theory can provide a useful framework to conceptualise problems relating to the self and interpersonal functioning. An examination of current attachment style can indicate how someone tends to see themselves and relate to others, and how this may influence functioning in later in life [53]. Different combinations of negative and positive models of self and of others generate four adult attachment styles i.e., secure, preoccupied, fearful and dismissive attachment, that can be dimensionally rated as more or less applicable to the individual [53–55]. Individuals manifesting a predominantly secure attachment style tend to see themselves as worthy of love and others as capable of fulfilling their needs in relationships [53]. Within the therapeutic context, attachment security has been shown to facilitate a positive treatment outcome [56]. The preoccupied attachment style is comprised of a negative model of self and a positive model of others, such that individuals manifesting a predominantly preoccupied style see themselves as unworthy of love, and are dependent on valued others to compensate for this lack of self-worth [53]. The fearful avoidant attachment style also features a negative model of self, but alongside a negative model of others [53]. It is thought that this distinct negative self-negative other profile creates conflict for the individual – whilst they desire relationships to compensate for chronically low self-worth, their belief that others cannot be trusted, leads to avoidance of relationships out of fear of being rejected. Fearful attachment, like the preoccupied style, has also been linked to poorer outcome from psychotherapy (e.g. [57]). Finally, the dismissive attachment style features a positive self model combined with a negative model of others. People with this style typically view others as untrustworthy or unable to meet their needs.

It has been observed that people with NPD hold both positive and negative models of themselves and tend to distrust others [9, 26, 27, 58]. A negative model of self may be defended against in unhelpful ways (e.g. fantasy, avoidance, aggression [33]) resulting in disturbed relatedness to others. Research indicates that vulnerable narcissism is related to a preoccupation with relationships for fear of being rejected [27, 59–63]. Grandiose narcissism has been linked to more secure and more dismissive attachment [64, 65]. However, here again, findings regarding grandiose narcissism and attachment have been less consistent with some studies finding no relation to attachment (e.g. [59, 62]). Inconsistencies in findings for grandiose narcissism with respect to shame as well as attachment may result from differences in how grandiose narcissism is operationalised e.g., as adaptive and agentic or maladaptive and antagonistic [16, 66]. Findings may also differ depending on whether the association between shame and grandiose narcissism is considered alongside vulnerable narcissism as overlap between grandiose and vulnerable narcissism has been observed both in clinical practice and in research [6, 67, 68]. Therefore, investigating vulnerable and grandiose narcissism simultaneously using multivariate models can account for their shared variance and allows one to discern the unique associations of each [69].

Furthermore, it should be noted that the way people relate to self and others may be in part traced back to early interactions with attachment figs [53, 70, 71].. These early ways of relating to self and others become internalised models of attachment that remain observable in adolescence and adulthood [53, 70, 71]. Similarly, narcissism traits and shame experiences may have their origin in early life [30, 72]. It is likely that a complex interplay between narcissism traits, shame experiences and attachment styles exists throughout life. However, from a therapeutic perspective, we seek to investigate the mechanism by which young people presenting with pathological narcissistic traits may experience intrapersonal and interpersonal difficulties. Therefore, this study seeks to examine whether shame is a mediator by which pathological grandiose and vulnerable narcissism relates to more negative models of self and others (expressed as current attachment style). By studying young people (i.e., late adolescence and emerging adulthood), we aim to capture a period within which pathological narcissism traits may be particularly relevant, given the high incidence of emerging mental health problems and opportunities for early intervention, coinciding with the formation of adult attachment styles and associated personality vulnerabilities [7, 12, 73–75].

We hypothesize that people higher in pathological narcissism, particularly vulnerable narcissism, will have greater experiences of shame and thereby have a negative model of self and others (preoccupied and/or fearful attachment). To further shed light on the associations with grandiose narcissism, particularly in young people, we will test the same associations and mediation for grandiose narcissism as for vulnerable narcissism. However, as previous findings on grandiose narcissism are mixed, the expectations of which associations should arise are less clear. As described above, we distinguish between grandiose and vulnerable pathological narcissism, and simultaneously assess their associations in a multivariate model to account for their shared variance. Finally, previous research indicate that certain facets of narcissism are specifically related to shame (e.g., contingent self-esteem, devaluing, grandiose fantasies). We will therefore explore in additional analyses how the different facets of narcissism (irrespective of grandiose or vulnerable narcissism) relate to shame and attachment experiences.

## Materials and methods

### Participants and procedure

Participants (*N* = 348, 78% women) in this study were young people (*M* = 19.27 years (*SD* = 1.61, Range = 17–25)), see Table 1. We targeted persons in late adolescence (16–18 years) and emerging adulthood (18–25 years) to recruit our sample and used the definition of young people for this age period [7, 17, 73, 76]. A snowball method of recruitment was used, where notices for the study were provided to young people who had finished high school and were contemplating study at university. Those participating were further encouraged to let others know about the study. Most participants were born in Australia (*N* = 310, 90%) and came from a family where parents were not divorced, separated or widowed (*N* = 267, 77%). Participants reported an average level but broad range of trait self-esteem (M = 30.33, SD = 5.83, Range = 10–40), comparable to those found in other general population samples [77]. Some participants reported having been diagnosed in their life with a mental health condition (*N* = 38, 11%) with depression and anxiety as the most commonly reported diagnoses.

**Table 1:** Demographic information on the sample (*N* = 348)

Demographic	N (%)/M (SD)
Gender	
- Female	270 (77.6%)
- Male	78 (22.4%)
Age	*M* = 19.27 (SD = 1.61)
Education	
- Completed high school	337 (96.8%)
- Completed Vocational college or training	11 (3.2%)
Marital status participant	
- Never married	325 (93.4%)
- Married	2 (0.6%)
- Widowed	1 (0.3%)
- Divorced or separated	20 (5.7%)
- Living together	0 (0%)
Family situation parents	
- Separated	12 (3.4%)
- Divorced	57 (16.4%)
- Widowed	12 (3.4%)
- Not separated, divorced or widowed	267 (76.7%)
Lifetime diagnosis	38 (10.9%)
Trait self-esteem	*M* = 30.33 (SD = 5.83)

The study received ethical approval from the Institutional Review Board of the University of Wollongong, Australia (HE10/370). Participants provided informed consent prior to participating. Participants volunteered their time (approx. 30 min) to participate in the study. Participants completed an online assessment module via a secure website. The data were checked for flat responses resulting in the exclusion of one participant resulting in the sample described above (*N* = 348). Part of this sample has been described in a separate paper [19].

### Measures

#### Pathological narcissism inventory

Grandiose and vulnerable narcissism traits were measured using the Pathological Narcissism Inventory [PNI; 29]. The PNI contains 52-items that are rated on a 6-point Likert scale ranging from not at all like me (0) to very much like me (5). Psychometric qualities of this instrument have been established [10, 29]. Grandiose narcissism was calculated as the mean score of the items of three subscales (Grandiose Fantasy (*α* = 0.86), Exploitativeness (*α* = 0.76) and Self-Sacrificing Self-Enhancement (*α* = 0.77)). Vulnerable narcissism was calculated as the mean score of the items of four subscales (Contingent Self-Esteem (*α* = 0.92), Hiding the Self (*α* = 0.81), Devaluing (*α* = 0.85) and Entitlement Rage (*α* = 0.85)) [10]. Grandiose (*α* = 0.87) and vulnerable (*α* = 0.94) narcissm showed good internal consistency in this study. For means, standard deviations and correlations, see Table 2.

**Table 2:** Means, standard deviations, and correlations with confidence intervals

Variable	*M*	*SD*	1	2	3	4	5	6
1. Grandiose narcissism	2.77	0.76						
2. Vulnerable narcissism	2.22	0.82	.65**					
			[.59, .71]					
3. Secure attachment	55.57	25.29	−.09	−.36**				
			[−.19, .02]	[−.44, −.26]				
4. Dismissive-avoidant attachment	53.22	25.42	.09	.06	−.03			
			[−.01, .20]	[−.05, .16]	[−.13, .08]			
5. Preoccupied attachment	37.39	26.16	.25**	.34**	−.06	−.12*		
			[.15, .35]	[.24, .43]	[−.17, .04]	[−.23, −.02]		
6. Fearful-avoidant attachment	49.14	30.48	.17**	.37**	−.48**	.16**	.23**	
			[.06, .27]	[.27, .46]	[−.56, −.40]	[.06, .26]	[.13, .33]	
7. Experience of shame	2.23	0.60	.30**	.50**	−.32**	.02	.29**	.34**
			[.20, .40]	[.41, .58]	[−.41, −.21]	[−.09, .13]	[.19, .39]	[.24, .43]

Note. M and SD are used to represent mean and standard deviation, respectively. Values in square brackets indicate the 95% confidence interval

* *p*-values are denoted as follows: * indicates *p* < .05. ** indicates *p* < .01

#### Relationship questionnaire

The Relationship Questionnaire (RQ) [53] asked participants to rate four paragraphs that describe secure, fearful, preoccupied and dismissive attachment style on a scale of not at all like me (0) to very much like me (100). The RQ was treated dimensionally producing a score for each of the four styles per participant. The four attachment styles represent different combinations of positive and negative model of self and others. A negative model of self is present in fearful and preoccupied attachment. A negative model of other is present in dismissive and fearful attachment. Secure attachment is characterized as a positive model of self and other. The RQ is widely used and has been validated against other attachment measures and differentially predicts treatment outcomes (e.g. [57]).

#### Experience of shame scale

The Experience of Shame Scale (ESS) assessed shame in three domains, i.e. experiencing shame about one’s character, behaviour and body [78]. Each of the domains is measured on a feeling, cognitive (concern for others opinion) and behavioural (avoidance) component. The ESS contains 25 items rating experiences in the past year on a 4-point scale ranging from not at all (1) to very much (4). The total score showed good internal consistency (*α* = 0.95).

### Statistical analysis

We conducted a mediation analysis to test whether the experience of shame mediates the relation between grandiose and vulnerable narcissism on the one hand and the four attachment styles on the other hand. We used the Lavaan package (version 0.6–3) in R (3.6.0) [79] to define a path analysis model where the direct effect of vulnerable and grandiose narcissism on the four attachment styles was simultaneously assessed, as well as the mediating (indirect) effect via the experience of shame. In this multivariate model, grandiose and vulnerable narcissism were allowed to correlate to take their shared variance into account [69]. The total effect was defined as the sum of the direct and indirect effect of vulnerable and grandiose narcissism on attachment styles. For inference on the parameter estimates we used the maximum likelihood estimation with bootstrapped standard errors (5000 bootstraps) [80]. There were missing data for 38 participants on the experience of shame scale, therefore the case-wise maximum likelihood estimation was used [79, 81]. All variables were standardized before analysis. Simulation studies have shown that a minimum sample size of *N* = 300 is sufficient to estimate small, medium and large indirect effects in a path analysis model with a power level of > 0.80 [82].

We performed an exploratory analysis where we repeated the mediation analysis as described above but replaced grandiose and vulnerable narcissism with the facet level subscales of narcissism traits. All seven PNI subscales were entered simultaneously as predictors.

## Results

### Pathological narcissism and attachment

Vulnerable narcissism is related to less positive and more negative models of self, i.e., a less secure (*b* = − 0.43, *SE* = 0.07, *p* < .001), more preoccupied (*b* = 0.26, *SE* = 0.08, *p* = .002) and more fearful attachment (*b* = 0.33, *SE* = 0.07, *p* < .001). Grandiose narcissism was related to more secure attachment (*b* = 0.24, *SE* = 0.06, *p* < .001). There was no relation between grandiose narcissism and dismissive attachment (*b* = 0.13, *SE* = 0.07, *p* = .069). For all parameter estimates, see Supplementary Table 1.

### Pathological narcissism and shame

Vulnerable narcissism was related to greater experiences of shame (*b* = 0.57, *SE* = 0.07, *p* < .001). Grandiose narcissism was not related to the experience of shame (*b* = − 0.07, *SE* = 0.07, *p* = .332).

### Shame as mediator for vulnerable narcissism

**Fig. 1:**
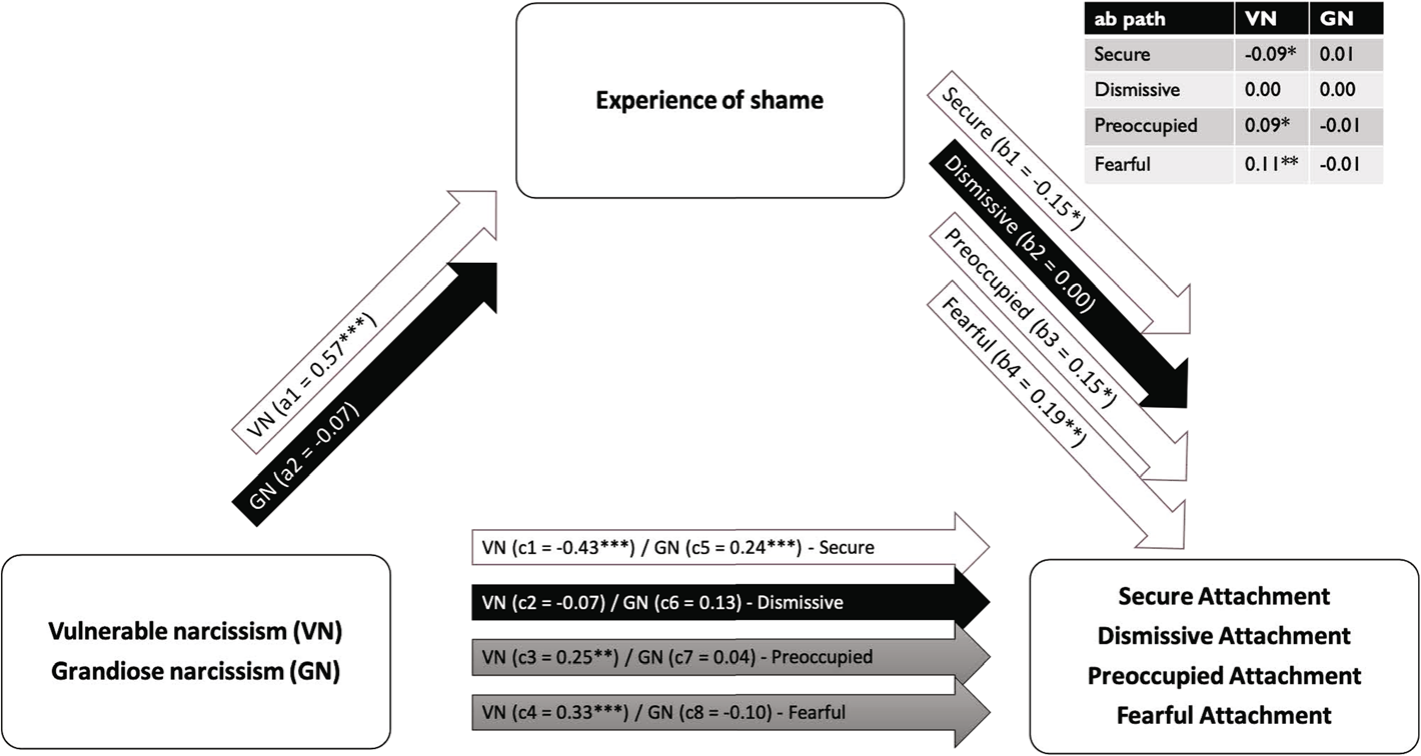
Results of mediation analysis of the experience of shame mediating between pathological narcissism and models of self and others

The experience of shame was related to less positive and more negative models of self, i.e. a less secure (*b* = − 0.15, *SE* = 0.06, *p* = .020), more preoccupied (*b* = 0.15, *SE* = 0.06, *p* = .016) and more fearful attachment (*b* = 0.19, *SE* = 0.06, *p* = .001), see Fig. 1 and Supplementary Table 1.

Shame mediated the relation between vulnerable narcissism and more negative models of self, i.e. fearful attachment (negative model of self and others) (*b* = 0.110, *SE* = 0.04, *p* = .003), preoccupied attachment (*b* = 0.09, *SE* = 0.04, *p* = .024), and (less) secure attachment (*b* = − 0.09, *SE* = 0.04, *p* = .022), see Fig. 1 and Supplementary Table 1.

### Grandiose narcissism

Shame did not mediate the relation between grandiose narcissism and any form of attachment, see Supplementary Table 1.

### Exploratory analysis of mediation at facet level

The exploratory analysis revealed that mainly contingent self-esteem was related to insecure attachment and shame, see Supplementary Table 2. Shame mediated between contingent self-esteem and fearful attachment (*b* = 0.11, *SE* = 0.04, *p* = .008).

## Discussion

People with more pathological narcissistic traits may have more maladaptive ways of relating to themselves and others. In this study, we found support for the hypothesis that the experience of shame acts a mechanism by which pathological narcissism traits (specifically vulnerable narcissism) relate to more negative models of self and more difficulty relating to others (i.e. less secure, more preoccupied and more fearful self-reported attachment).

Young people higher in vulnerable narcissism traits reported a need for being close to others but also a difficulty trusting others. Exploratory analyses indicated that three facets of vulnerable narcissism, namely contingent self-esteem, hiding the self and devaluing self and others, were mainly related to self and interpersonal difficulties. Previous studies in adults have indicated that vulnerable narcissism relates to more maladaptive interpersonal styles [59–62, 64]. Studies in adults and adolescents with NPD indicate that these interpersonal styles may not so much consist of avoidance but more of anxiety and preoccupation with relationships while also being dismissive of others [63, 65, 71]. Studies in adolescents show that vulnerable narcissism is associated with higher pro-active aggression and social stress [7] and that peers perceive adolescents higher in narcissism as more antagonistic [83]. It may be that young people express the need to be close to others, while perceiving others as not willing or able to reciprocate, in an antagonistic manner.

We further found that vulnerable narcissism, particularly contingent self-esteem (i.e., being dependent on others for self-esteem), is related to more experiences of shame. Although increases in shame experiences are in part normative in adolescence and emerging adulthood [84], it appears that young people with narcissism traits experience shame to a greater degree. In line with our finding, a recent study in (emerging) adults indicated that vulnerable narcissism, particularly contingent self-esteem, was related to experiences of shame on a day-to-day basis [49]. Moreover, our finding is in line with the theory that people higher in narcissism have developed a weaker sense of self that relies more heavily on how other people view them (‘objective self’) at the cost of relying less on inner experiences (‘subjective self’) [25, 30, 85]. However, a weak sense of self (i.e. a less integrated self) may relate to people being more prone to experiencing shame [86], as we have observed in this study. Shame highlights undesirable, stable and global attributes of the self [37, 38]. Since shame involves evaluating the self *as a whole* more negatively, rather than focussing on specific aspects of the self (e.g., feeling guilty about a certain behaviour) [38, 87], the experience of shame can highlight a discrepancy between the ideal and actual self [84]. This process may contribute to developing a more negative model of self by inhibiting the integration and strengthening of the self [33, 88].

Indeed, our findings indicate that shame mediated the relation between vulnerable narcissism traits and preoccupied, fearful and less secure attachment (i.e., difficulty relating to self and others). In other words, more negative models of self can be partially explained by experiencing shame. Moreover, shame may give rise to more interpersonal difficulties such as clinging to others but also disengaging from others [30, 36, 89–91]. Research indicates that for adolescents, shame may be related to less pro-social behaviours and is even causally related to greater hostility [92, 93]. More aggression has also been observed in children with narcissistic traits who were shamed [46]. Although, further research should further clarify the direction of effects, it could be that young people who rely more on others for their self-worth may be more prone to experiencing shame. Young people responding to shame in a hostile and antagonistic manner may further impede self and interpersonal functioning.

Young people higher in grandiose narcissism self-reported a more positive model of self and others (i.e. more secure), consistent with previous research in adults [61, 63, 64]. Although previous research has observed a relation between grandiose narcissism and dismissive attachment, in this study we did not observe this relation [64, 65]. Moreover, there was no relation between grandiose narcissism and the experience of shame. As we analysed grandiose narcissism simultaneously with vulnerable narcissism in a multivariate model, it could be that we did not observe a unique relationship between grandiose narcissism and more maladaptive attachment or shame. Different configurations of the narcissism facets that compose grandiose and vulnerable narcissism have been proposed [67]. However, our exploratory analysis on the facet level indicates that facets typically associated with grandiose narcissism did not relate to shame or attachment, except for self-sacrificing self-enhancement which related to more secure and less fearful attachment.

It could be that more maladaptive grandiose traits were not captured as our sample of young people was relatively healthy or because certain narcissism traits, such as the idea of omnipotence, could be adaptive in adolescence and emerging adulthood [15]. Other studies in adolescence, however, have related grandiose narcissism traits to psychopathology [94, 95]. It is thus unclear whether grandiose narcissism being unrelated to shame and insecure attachment in this study is an adaptive form of narcissism [66, 96] or whether the relation between grandiose narcissism and shame and attachment experiences might be challenging to capture with self-report measures [31]. Research using measures of implicit self-esteem and implicit shame have found a relation with grandiose narcissism [34, 97, 98]. Moreover, informant reports of narcissism traits which partially overlap with self-reports, also highlight a dismissive yet affirmation needing stance towards others as a feature of grandiose narcissism [99–101]. Why those with higher grandiosity may potentially dismiss emotions such as shame and attachment anxieties requires further research, but it may be due to a self-presentation of a “false” positive self as described in the clinical literature [102].

In sum, for vulnerable narcissism, the experience of shame relates to evaluating the self more negatively and struggling between wanting to be close to others but also not trusting others. It should be also considered that both grandiose and vulnerable narcissistic traits can be present within the same person [6, 68]. Research in adolescents has shown that NPD can be distinguished in three subtypes: predominantly grandiose/malignant, fragile and high-functioning/exhibitionistic [95, 103]. However, feelings of grandiosity and vulnerability may still fluctuate on a state basis within the individual [104]. Moreover, particularly when grandiose narcissism is higher (more pathological) grandiose and vulnerable narcissism may co-occur blending in their antagonistic core [105, 106], posing challenges for the therapeutic alliance and treatment outcomes [3, 5, 107, 108]. Fostering self-compassion in treatment, including with young people, may counter the disruptive effects of shame by allowing one’s needs and sense of self, including negative aspects of the self, to be experienced [26, 109]. The challenge here is that feelings of shame may relate to less self-compassion or even fear thereof in adolescents with narcissistic traits [110, 111] and that therapists may show less warm responses to adolescents presenting with pathological narcissistic traits [103]. In clinical practice, it is important to be aware of emotional reactions to clients with narcissistic traits to be able to foster self-compassion [103].

### Strengths and limitations

A limitation was that we only sampled the young participants once through a cross-sectional survey, and therefore cannot draw conclusions on the direction of effects proposed in this mediation analyses. Thus, it may also be possible that shame plays a role in the onset of more vulnerable narcissistic traits [30] or that the relationship might co-occur. This work may contribute to longitudinal and experimental approaches that are needed in this area [8]. Strengths of this study included the large sample allowing confidence in the tests for mediation and estimating the effects of vulnerable and grandiose narcissism traits simultaneously allowing for a more ecological valid model to be obtained. Moreover, the sample consisted of young people who were at a vulnerable age for developing personality disorders such as NPD [7, 17, 73]. However, it was a relatively healthy sample from the general population and replication in a clinical sample is warranted, particularly to shed more light on the associations involving grandiose narcissism.

## Conclusion

People with vulnerable narcissism traits tended to report more shame, and struggled to be close to others. It may be that shame experiences highlight a discrepancy between the ideal and actual self that may contribute to a more insecure attachment style. Those with more grandiose narcissism traits we think were more likely to dismiss negative emotions or attachments. Clinically, a good working alliance and fostering self-compassion may counter some negative effects of shame, strengthening a person’s internal security and positive attachments with others.

## Supplementary Information

**Additional file 1: Supplementary Table S1.** Parameters estimates of direct, indirect and total effects of mediation model. **Supplementary Table S2.** Parameters estimates of direct, indirect and total effects of mediation model by facets of pathological narcissism traits.

## Data Availability

The dataset analysed during the current study does not have clearance to be made publicly available but the analysis script can be found on Open Science Framework (10.17605/OSF.IO/UN7DP).

## References

[1] Winsper C, Bilgin A, Thompson A, Marwaha S, Chanen AM, Singh SP, Wang A, Furtado V (2020). The prevalence of personality disorders in the community: a global systematic review and meta-analysis. Br J Psychiatry.

[2] Biskin RS (2015). The lifetime course of borderline personality disorder. Can J Psychiatr.

[3] Day NJS, Bourke M, Townsend ML, Grenyer BFS (2019). Pathological narcissism: a study of burden on partners and family. J Personal Disord.

[4] Cristea IA, Gentili C, Cotet CD, Palomba D, Barbui C, Cuijpers P (2017). Efficacy of psychotherapies for borderline personality disorder: a systematic review and meta-analysis. JAMA Psychiatry.

[5] King RM, Grenyer BFS, Gurtman CG, Younan R (2020). A clinician's quick guide to evidence-based approaches: narcissistic personality disorder. Clin Psychol.

[6] Grenyer BFS: Historical overview of pathological narcissism. In: *Understanding and treating pathological narcissism.* edn. Edited by Ogrodniczuk JS. Washington, DC: American Psychological Association; 2013: 15–26.

[7] Barry CT, Kauten RL (2014). Nonpathological and pathological narcissism: which self-reported characteristics are Most problematic in adolescents?. J Pers Assess.

[8] Thomaes S, Bushman BJ, Orobio de Castro B, Stegge H (2009). What makes narcissists bloom? A framework for research on the etiology and development of narcissism. Dev Psychopathol.

[9] Krizan Z, Herlache AD (2018). The narcissism Spectrum model: a synthetic view of narcissistic personality. Personal Soc Psychol Rev.

[10] Wright AGC, Lukowitsky MR, Pincus AL, Conroy DE (2010). The higher order factor structure and gender invariance of the pathological narcissism inventory. Assessment.

[11] Caligor E, Levy KN, Yeomans FE (2015). Narcissistic personality disorder: diagnostic and clinical challenges. Am J Psychiatry.

[12] Hill PL, Lapsley DK: Adaptive and maladaptive narcissism in adolescent development. In: *Narcissism and Machiavellianism in Youth: Implications for the Development of Adaptive and Maladaptive Behavior.* edn. Edited by Barry CT, Kerig PK, Stellwagen KK, Barry TD: American Psychological Association; 2011: 89–105.

[13] Carlson KS, Gjerde PF (2009). Preschool personality antecedents of narcissism in adolescence and young adulthood: a 20-year longitudinal study. J Res Pers.

[14] Cramer P (1995). Identity, narcissism, and defense mechanisms in late adolescence. J Res Pers.

[15] Aalsma MC, Lapsley DK, Flannery DJ (2006). Personal fables, **,** And Adolescent Adjustment narcissism. Psychol Sch.

[16] Bleiberg E (1994). Normal and pathological narcissism in adolescence. Am J Psychother.

[17] Sharp C, Wall K (2018). Personality pathology grows up: adolescence as a sensitive period. Curr Opin Psychol.

[18] Cramer P (2017). Identity change between late adolescence and adulthood. Pers Individ Dif.

[19] van Schie CC, Jarman HL, Huxley E, Grenyer BFS (2020). Narcissistic traits in young people: understanding the role of parenting and maltreatment. Borderline Personality Disorder and Emotion Dysregulation.

[20] Brummelman E, Thomaes S, Nelemans SA (2015). Orobio de Castro B, Overbeek G, Bushman BJ: **origins of narcissism in children**. Proc Natl Acad Sci U S A.

[21] Horton RS: Parenting as a Cause of Narcissism: Empirical Support for Psychodynamic and Social Learning Theories. In: *The Handbook Of Narcissism And Narcissistic Personality Disorder Theoretical Approaches, Empirical Findings, And Treatments.* edn. Edited by Campbell DW, Miller JD. Hoboken, New Jersey: John Wiley & Sons, Inc; 2011: 181–190.

[22] Kernis MH (2003). TARGET ARTICLE: toward a conceptualization of optimal self-esteem. Psychol Inq.

[23] Morf CC, Rhodewalt F (2001). Unraveling the paradoxes of narcissism: a dynamic self-regulatory processing model. Psychol Inq.

[24] Rothstein A (1986). The theory of narcissism: an object-relations perspective. In: *Essential Papers on Narcissism.* Edn. Edited by Morrison AP.

[25] Rhodewalt F, Madrian JC, Cheney S (1998). Narcissism, self-knowledge organization, and emotional reactivity: the effect of daily experiences on self-esteem and affect. Personal Soc Psychol Bull.

[26] Kramer U, Pascual-Leone A, Rohde KB, Sachse R (2018). The role of shame and self-compassion in psychotherapy for narcissistic personality disorder: an exploratory study. Clinical Psychology and Psychotherapy.

[27] Miller JD, Lynam DR, Hyatt CS, Campbell WK (2017). Controversies in narcissism. Annu Rev Clin Psychol.

[28] McWilliams N (2011). **Psychoanalytic diagnosis. Understanding personality structure in the clinical process.**, 2nd edn.

[29] Pincus AL, Ansell EB, Pimentel CA, Cain NM, Wright AGC, Levy KN (2009). Initial construction and validation of the pathological narcissism inventory. Psychol Assess.

[30] Broucek FJ (1982). Shame and its relationship to early narcissistic developments. Int J Psychoanal.

[31] Bosson JK, Prewitt-Freilino JL: Overvalued and ashamed: considering the roles of self-esteem and self-conscious emotions in covert narcissism. In: *The self-conscious emotions: Theory and research.* 2 edn. Edited by Tracy JL, Robins RW, Tangney JP. New York: Guilford; 2007.

[32] Ronningstam E (2016). Pathological narcissism and narcissistic personality disorder: recent research and clinical implications. Curr Behav Neuroscience Rep.

[33] Schoenleber M, Berenbaum H. Shame regulation in personality pathology. J Abnorm Psychol. 2011. 10.1037/a0025281 21895346

[34] Ritter K, Vater A, Rusch N, Schroder-Abe M, Schutz A, Fydrich T, Lammers CH, Roepke S (2014). Shame in patients with narcissistic personality disorder. Psychiatry Res.

[35] Wright F (1989). Shame, guilt, narcissism and depression: correlates and sex differences. Psychoanal Psychol.

[36] Kemeny ME, Gruenewald TL, Dickerson SS (2004). Shame as the emotional response to threat to the social self: implications for behavior, physiology, and health. Psychol Inq.

[37] Tracy JL, Robins RW (2004). Putting the self into self-conscious emotions: a theoretical model. Psychol Inq.

[38] Niedenthal PM, Tangney JP, Gavanski I (1994). “if only I Weren’t” versus “if only I Hadn’t”: distinguishing shame and guilt in counterfactual thinking. J Pers Soc Psychol.

[39] Tangney JP, Stuewig J, Mashek DJ (2007). Moral emotions and moral behavior. Ann Rev Psychology.

[40] Beer JS, Heerey EA, Keltner D, Scabini D, Knight RT (2003). The regulatory function of self-conscious emotion: insights from patients with orbitofrontal damage. J Pers Soc Psychol.

[41] Shen L: The evolution of shame and guilt. PLoS One 2018, 13(7). 10.1371/journal.pone.0199448 PMC6040729 29995883

[42] Beer JS, Heerey EA, Keltner D, Scabini D, Knight RT (2003). The regulatory function of self-conscious emotion: insights from patients with orbitofrontal damage. J Pers Soc Psychol.

[43] de Hooge IE, Zeelenberg M, Breugelmans SM (2010). Restore and protect motivations following shame. Cognit Emot.

[44] Hejdenburg J, Andrews B (2011). The relationship between shame and different types of anger: a theory-based investigation. Pers Individ Dif.

[45] Tracey JL, Robins RW (2003). **"**Death of a (narcissistic) salesman:" An Integrative Model of Fragile Self-Esteem. Psychol Inq.

[46] Thomaes S, Bushman BJ, Stegge H, Olthof T (2008). Trumping shame by blasts of noise: narcissism, self-esteem, shame, and aggression in young adolescents. Child Dev.

[47] Cain NM, Pincus AL, Ansell EB (2008). Narcissism at the crossroads: phenotypic description of pathological narcissism across clinical theory, social/personality psychology, and psychiatric diagnosis. Clin Psychol Rev.

[48] Hibbard S (1992). Narcissism, shame, masochism, and object relations: an exploratory correlational study. Psychoanal Psychol.

[49] DiSarno M, Zimmerman J, Madeddu F, Casini E, Di Pierro R. Shame behind the corner? A daily diary investigation of pathological narcissism. J Res Pers. 2020;85.

[50] Freis SD, Brown AA, Carroll PJ, Arkin RM (2015). Shame, rage**,** And Unsuccessful Motivated Reasoning In Vulnerable Narcissism. J Soc Clin Psychol.

[51] Malkin ML, Barry CT, Zeigler-Hill V (2011). Covert narcissism as a predictor of internalizing symptoms after performance feedback in adolescents. Pers Individ Dif.

[52] Montebarocci O, Surcinelli P, Baldaro B, Trombini E, Rossi N (2004). Narcissism versus proneness to shame and guilt. Psychol Rep.

[53] Bartholomew K, Horowitz L (1991). Attachment styles among young adults: a test of a four-category model. J Pers Soc Psychol.

[54] Ravitz P, Maunder R, Hunter J, Sthankiya B, Lancee W (2010). Adult attachment measures: a 25-year review. J Psychosom Res.

[55] Fraley RC, Hudson NW, Heffernan ME, Segal N (2015). Are adult attachment styles categorical or dimensional? A Taxometric analysis of general and relationship-specific attachment orientations. J Pers Soc Psychol.

[56] Levy KN, Kivity Y, Johnson BN, Gooch CV (2018). Adult attachment as a predictor and moderator of psychotherapy outcome: a meta-analysis. J Clin Psychol.

[57] Reis S, Grenyer BF (2004). Fearful attachment, working Alliance and treatment response for individuals with major depression. Clinical Psychol Psychother.

[58] Jauk E, Benedek M, Koschutnig K, Kedia G, Neubauer AC (2017). Self-viewing is associated with negative affect rather than reward in highly narcissistic men: an fMRI study. Sci Rep.

[59] Miller JD, Hoffman BJ, Gaughan ET, Gentile B, Maples J, Keith Campbell W (2011). Grandiose and vulnerable narcissism: a nomological network analysis. J Pers.

[60] Otway LJ, Vignoles VL (2006). Narcissism and childhood recollections: a quantitative test of psychoanalytic predictions. Personal Soc Psychol Bull.

[61] Rohmann E, Neumann E, Herner MJ, Bierhoff H-W (2012). Grandiose and vulnerable narcissism. Eur Psychol.

[62] Smolewska K, Dion K (2005). Narcissism and adult attachment: a multivariate approach. Self Identity.

[63] Fossati A, Feeney J, Pincus A, Borroni S, Maffei C (2015). The structure of pathological narcissism and its relationships with adult attachment styles: a study of Italian nonclinical and clinical adult participants. Psychoanal Psychol.

[64] Dickinson KA, Pincus AL (2003). Interpersonal analysis of grandiose and vulnerable narcissism. J Personal Disord.

[65] Rosenstein DS, Horowitz HA (1996). Adolescent attachment and psychopathology. J Consult Clin Psychol.

[66] Back MD, Kufner ACP, Dufner M, Gerlach TM, Rauthmann JF, Denissen JJA (2013). Narcissistic admiration and rivalry: disentangling the bright and dark sides of narcissism. J Pers Soc Psychol.

[67] Weiss M, Fradkin I, Huppert JD. Modelling pathological narcissism using the brief PNI in terms of structure and convergent and divergent validity: a new perspective. Assessment. 2020:1–11. 10.1177/1073191120936354 32589049

[68] Pincus AL, Cain NM, Wright AGC (2014). Narcissistic grandiosity and narcissistic vulnerability in psychotherapy. Personal Disord Theory Res Treat.

[69] Edershile EA, Simms LJ, Wright AGC (2019). A multivariate analysis of the pathological narcissism Inventory’s Nomological network. Assessment.

[70] Cramer P (2019). Narcissism and attachment: the importance of early parenting. J Nerv Ment Dis.

[71] Neumann E (2017). Emotional abuse in childhood and attachment anxiety in adult romantic relationships as predictors of personality disorders. J Aggress Maltreat Trauma.

[72] Thomaes S, Brummelman E, Reijntjes A, Bushman BJ (2013). When Narcissus was a boy: origins, nature, and consequences of childhood narcissism. Child Dev Perspect.

[73] Rickwood DJ, Telford NR, Parker AG, Tanti CJ (2014). McGorry PD: **headspace — Australia's innovation in youth mental health: who are the clients and why are they presenting?**. Med J Aust.

[74] Sharp C (2020). Adolescent personality pathology and the alternative model for personality disorders: self development as nexus. Psychopathology.

[75] Cohen P, Crawford TN, Johnson JG, Kasen S (2005). The children in the community study of developmental course of personality disorder. J Personal Disord.

[76] Chopik WJ, Grimm KJ (2019). Longitudinal changes and historic differences in narcissism from adolescence to older adulthood. Psychol Aging.

[77] Schmitt DP, Allik J (2005). Simultaneous administration of the Rosenberg self-esteem scale in 53 nations: exploring the universal and culture-specific features of global self-esteem. J Pers Soc Psychol.

[78] Andrews B, Qian M, Valentine JD (2002). Predicting depressive symptoms with a new measure of shame: the experience of shame scale. Br J Clin Psychol.

[79] Rosseel Y (2012). Lavaan: an R package for structural equation modeling. J Stat Softw.

[80] Hayes AF (2009). Beyond baron and Kenny: statistical mediation analysis in the new millennium. Commun Monogr.

[81] Allison PD (2003). Missing data techniques for structural equation modeling. J Abnorm Psychol.

[82] Wolf EJ, Harrington KL, Clark SL, Miller MW (2013). Sample size requirements for structural equation models: an evaluation of power, Bias, and solution propriety. Educ Psychol Meas.

[83] Grafeman SJ, Barry CT, Marcus DK, Leachman LL (2013). Interpersonal perceptions of narcissism in an at-risk adolescent sample: a social relations analysis. J Res Adolesc.

[84] Reimer MS (1996). “Sinking into the ground”: the development and consequences of shame in adolescence. Dev Rev.

[85] Geukes K, Nestler S, Dufner M, Egloff B, Hutteman R, Kufner ACP, Denissen JJA, Back MD (2017). Puffed-up but shaky selves: state self-esteem level and variability in narcissists. J Pers Soc Psychol.

[86] Gramzow R, Tangney JP (1992). Proneness to shame and the Narcisstistic personality. Personal Soc Psychol Bull.

[87] Gruenewald TL, Kemeny ME, Aziz N, Fahey JL (2004). Acute threat to the social self: shame, social self-esteem, and cortisol activity. Psychosom Med.

[88] Cain NM, Pincus AL, Ansell EB (2008). Narcissism at the crossroads: phenotypic description of pathological narcissism across clinical theory, social/personality psychology, and psychiatric diagnosis. Clin Psychol Rev.

[89] Chen C, Hewitt PL, Flett GL (2015). Preoccupied attachment, need to belong, shame, and interpersonal perfectionism: an investigation of the perfectionism social disconnection model. Pers Individ Dif.

[90] Gross CA, Hansen NE (2000). Clarifying the experience of shame: the role of attachment style, gender, and investment in relatedness. Pers Individ Dif.

[91] Passanisi A, Gervasi AM, Madonia C, Guzzo G, Greco D (2015). Attachment, self-esteem and shame in emerging adulthood. Procedia - Social and Behavioral Sciences.

[92] Roos S, Hodges EVE, Salmivalli C (2014). Do guilt- and shame-proneness differentially predict prosocial, aggressive, and withdrawn behaviors during early adolescence?. Dev Psychol.

[93] Heaven PCL, Ciarrochi J, Leeson P (2009). The longitudinal links between shame and increasing hostility during adolescence. Pers Individ Dif.

[94] Xu X, Huebner ES, Tian L (2020). Profiles of narcissism and self-esteem associated with comprehensive mental health in adolescents. J Adolesc.

[95] Lapsley DK, Aalsma MC (2006). An empirical typology of narcissism and mental health in late adolescence. J Adolesc.

[96] Dashineau SC, Edershile EA, Simms LJ, Wright AGC (2019). Pathological narcissism and psychosocial functioning. Personal Disord Theory Res Treat.

[97] Bosson JK, Lakey CE, Campbell WK, Zeigler-Hill V, Jordan CH, Kernis MH (2008). Untangling the links between narcissism and self-esteem: a theoretical and empirical review. Soc Personal Psychol Compass.

[98] Jordan CH, Spencer SJ, Zanna MP, Hoshino-Browne E, Correll J (2003). Secure and defensive high self-esteem. J Pers Soc Psychol.

[99] Day NJS, Townsend ML, Grenyer BFS (2020). Living with pathological narcissism: a qualitative study. Borderline Personality Disorder and Emotion Dysregulation.

[100] Maples-Keller JL, Miller JD (2018). Insight and the dark triad: comparing self- and meta-perceptions in relation to psychopathy, narcissism, and Machiavellianism. Personal Disord Theory Res Treat.

[101] Lukowitsky MR, Pincus AL (2013). Interpersonal perception of pathological narcissism: a social relations analysis. J Pers Assess.

[102] Winnicott DW (1965). Ego distortion in terms of true and false self. In: The Maturational Processes and the Facilitating Environment: studies in the theory of emotional development. Edn.

[103] Tanzilli A, Gualco I (2020). Clinician emotional responses and therapeutic Alliance when treating adolescent patients with narcissistic personality disorder subtypes: a clinically meaningful empirical investigation. J Personal Disord.

[104] Oltmans JR, Widiger TA (2018). Assessment of fluctuation between grandiose and vulnerable narcissism: development and initial validation of the FLUX scales. Psychol Assess.

[105] Jauk E, Kaufman SB. The higher the score, the darker the Core: the nonlinear association between grandiose and vulnerable narcissism. Front Psychol. 2018;9. 10.3389/fpsyg.2018.01305. 10.3389/fpsyg.2018.01305 PMC6088174 30150950

[106] Jauk E, Weigle E, Lehman K, Benedek M, Neubauer AC. The relationship between grandiose and vulnerable (hypersensitive) narcissism. Front Psychol. 2017;8. 10.3389/fpsyg.2017.01600. 10.3389/fpsyg.2017.01600 PMC5601176 28955288

[107] Ogrodniczuk JS, Piper WE, Joyce AS, Steinberg PI, Duggal S (2009). Interpersonal problems associated with narcissism among psychiatric outpatients. J Psychiatr Res.

[108] WHJ M (2005). Ann Am Psychother Assoc.

[109] Barry CT, Loflin DC, Doucette H (2015). Adolescent self-compassion: associations with narcissism, self-esteem, aggression, and internalizing symptoms in at-risk males. Pers Individ Dif.

[110] Castilho P, Carvalho SA, Marques S, Pinto-Gouveia J (2017). Self-compassion and emotional intelligence in adolescence: a multigroup mediational study of the impact of shame memories on depressive symptoms. J Child Fam Stud.

[111] Xavier A, Pinto-Gouveia J, Cunha M (2016). Non-suicidal self-injury in adolescence: the role of shame, self-criticism and fear of self-compassion. Child Youth Care Forum.

[112] World Medical Association (2013). World medical association declaration of Helsinki ethical principles for medical research involving human subjects. JAMA: Journal of the American Medical Association.

