# Supplementary material for: Narcissistic traits in young people and how experiencing shame relates to current attachment challenges
Source: BMC Psychiatry. 2021 May 11;21:246. doi: 10.1186/s12888-021-03249-4 (PMC8112045; doi:10.1186/s12888-021-03249-4)
Supplement: Supplementary file 1 — Additional file 1: Supplementary Table S1. Parameters estimates of direct, indirect and total effects of mediation model. Supplementary Table S2. Parameters estimates of direct, indirect and total effects of mediation model by facets of pathological narcissism traits. [file 12888_2021_3249_MOESM1_ESM.pdf]

Narcissistic traits in young people and how experiencing shame relates to current attachment challenges

Charlotte C. van Schie[1], Heidi L. Jarman[2], Samantha Reis[1], Brin F. S. Grenyer[1]*

[1]Illawarra Health and Medical Research Institute and the School of Psychology, University of Wollongong, Wollongong, Australia

Address: Northfields Avenue, University of Wollongong, NSW 2522, Australia

[2] The Reflective Space: Clinical Psychology & Psychotherapy Services

Address: PO Box 778 Milsons Point, NSW 1565, Australia

***Correspondence:**

Brin Grenyer

E-mail address: grenyer@uow.edu.au

Address: Northfields Avenue, University of Wollongong NSW 2522, Australia

Supplemental information

Supplementary Table 1

Parameters estimates of direct, indirect and total effects of mediation model.

Regressions:

Outcome	Predictor	Path	Parameter estimate	Standard error	z-value	p-value	95% CI Lower limit	95% CI Upper limit
Secure	Vulnerable narcissism	c1	-0.433	0.074	-5.861	0.000	-0.579	-0.288
	Grandiose narcissism	c5	0.236	0.062	3.788	0.000	0.119	0.364
Dismissive	Vulnerable narcissism	c2	-0.068	0.083	-0.827	0.408	-0.228	0.100
	Grandiose narcissism	c6	0.134	0.074	1.820	0.069	-0.010	0.285
Preoccupied	Vulnerable narcissism	c3	0.255	0.081	3.171	0.002	0.097	0.419
	Grandiose narcissism	c7	0.041	0.069	0.604	0.546	-0.099	0.175
Fearful	Vulnerable narcissism	c4	0.327	0.073	4.461	0.000	0.183	0.471
	Grandiose narcissism	c8	-0.102	0.068	-1.497	0.134	-0.235	0.030
Shame	Vulnerable narcissism	a1	0.565	0.065	8.706	0.000	0.434	0.692
	Grandiose narcissism	a2	-0.066	0.068	-0.969	0.332	-0.195	0.066
Secure	Shame	b1	-0.150	0.064	-2.335	0.020	-0.273	-0.029
Dismissive	Shame	b2	0.004	0.076	0.051	0.959	-0.151	0.152
Preoccupied	Shame	b3	0.152	0.063	2.412	0.016	0.029	0.274
Fearful	Shame	b4	0.195	0.061	3.217	0.001	0.079	0.318

Indirect effect:		Path	Parameter estimate	Standard error	z-value	p-value	95% CI Lower limit	95% CI Upper limit
Vulnerable narcissism - Shame	Secure	a1b1	-0.085	0.037	-2.287	0.022	-0.155	-0.017
Vulnerable narcissism - Shame	Dismissive	a1b2	0.002	0.043	0.051	0.959	-0.088	0.085
Vulnerable narcissism - Shame	Preoccupied	a1b3	0.086	0.038	2.250	0.024	0.015	0.164
Vulnerable narcissism - Shame	Fearful	a1b4	0.110	0.038	2.927	0.003	0.042	0.191

Grandiose narcissism - Shame	Secure	a2b1	0.010	0.012	0.855	0.393	-0.011	0.035
Grandiose narcissism - Shame	Dismissive	a2b2	0.000	0.007	-0.035	0.972	-0.015	0.018
Grandiose narcissism - Shame	Preoccupied	a2b3	-0.010	0.012	-0.837	0.403	-0.038	0.010
Grandiose narcissism - Shame	Fearful	a2b4	-0.013	0.014	-0.902	0.367	-0.044	0.014
Total effect:			Parameter estimate	Standard error	z-value	p-value	95% CI Lower limit	95% CI Upper limit
Vulnerable narcissism - Shame	Secure	c1+a1b1	-0.517	0.064	-8.055	0.000	-0.643	-0.390
Vulnerable narcissism - Shame	Dismissive	c2+a1b2	-0.066	0.070	-0.943	0.346	-0.207	0.076
Vulnerable narcissism - Shame	Preoccupied	c3+a1b3	0.341	0.070	4.904	0.000	0.207	0.483
Vulnerable narcissism - Shame	Fearful	c4+a1b4	0.437	0.063	6.909	0.000	0.311	0.561
Grandiose narcissism - Shame	Secure	c5+a2b1	0.246	0.064	3.849	0.000	0.124	0.375
Grandiose narcissism - Shame	Dismissive	c6+a2b2	0.134	0.074	1.820	0.069	-0.010	0.287
Grandiose narcissism - Shame	Preoccupied	c7+a2b3	0.031	0.069	0.457	0.647	-0.109	0.163
Grandiose narcissism - Shame	Fearful	c8+a2b4	-0.114	0.069	-1.658	0.097	-0.250	0.019
Covariances:			Parameter estimate	Standard error	z-value	p-value	95% CI Lower limit	95% CI Upper limit
Vulnerable narcissism	Grandiose narcissism		0.655					
Secure	Dismissive		-0.039	0.054	-0.716	0.474	-0.144	0.069
Secure	Preoccupied		0.078	0.046	1.695	0.090	-0.011	0.171
Secure	Fearful		-0.316	0.050	-6.345	0.000	-0.410	-0.214
Dismissive	Preoccupied		-0.134	0.054	-2.490	0.013	-0.238	-0.029
Dismissive	Fearful		0.164	0.053	3.109	0.002	0.062	0.268
Preoccupied	Fearful		0.079	0.047	1.684	0.092	-0.013	0.168

Note: Standardised variables are used in analyses therefore the parameter estimates are standardised parameters.

Supplementary Table 2

Parameters estimates of direct, indirect and total effects of mediation model by facets of pathological narcissism traits.

Regressions:

Outcome	Predictor	Path	Parameter estimate	Standard error	z-value	p-value	95% CI Lower limit	95% CI Upper limit
Secure	CSE	**c1**	**-0.243**	**0.092**	**-2.630**	**0.009**	**-0.424**	**-0.058**
	ENTR	c2	0.057	0.082	0.702	0.483	-0.098	0.225
	HS	**c3**	**-0.269**	**0.065**	**-4.124**	**0.000**	**-0.395**	**-0.139**
	DEV	c4	-0.138	0.075	-1.841	0.066	-0.283	0.011
	GF	c5	0.015	0.062	0.238	0.812	-0.110	0.140
	EXP	c6	0.060	0.056	1.066	0.286	-0.051	0.172
	SSSE	**c7**	**0.237**	**0.067**	**3.553**	**0.000**	**0.108**	**0.372**
Dismissive	CSE	**c8**	**-0.521**	**0.090**	**-5.794**	**0.000**	**-0.690**	**-0.335**
	ENTR	c9	0.143	0.083	1.736	0.083	-0.023	0.306
	HS	**c10**	**0.261**	**0.063**	**4.152**	**0.000**	**0.132**	**0.380**
	DEV	c11	0.086	0.071	1.224	0.221	-0.050	0.224
	GF	c12	0.091	0.065	1.400	0.161	-0.037	0.216
	EXP	c13	0.042	0.062	0.679	0.497	-0.080	0.160
	SSSE	c14	-0.042	0.068	-0.618	0.536	-0.177	0.093
Preoccupied	CSE	**c15**	**0.303**	**0.089**	**3.395**	**0.001**	**0.133**	**0.483**
	ENTR	c16	0.018	0.088	0.206	0.837	-0.163	0.186
	HS	c17	-0.115	0.066	-1.737	0.082	-0.245	0.013
	DEV	c18	0.055	0.082	0.678	0.498	-0.103	0.216
	GF	c19	0.027	0.072	0.380	0.704	-0.117	0.168

	EXP	c20	0.015	0.060	0.260	0.795	-0.100	0.137
	SSSE	c21	0.052	0.073	0.717	0.473	-0.086	0.197
Fearful	CSE	c22	0.119	0.087	1.361	0.173	-0.058	0.284
	ENTR	c23	-0.140	0.081	-1.720	0.086	-0.300	0.023
	HS	**c24**	**0.355**	**0.053**	**6.722**	**0.000**	**0.249**	**0.457**
	DEV	**c25**	**0.143**	**0.073**	**1.959**	**0.050**	**0.001**	**0.287**
	GF	c26	0.015	0.063	0.247	0.805	-0.106	0.141
	EXP	c27	-0.019	0.056	-0.338	0.735	-0.127	0.091
	SSSE	**c28**	**-0.144**	**0.065**	**-2.208**	**0.027**	**-0.273**	**-0.017**
Shame	CSE	**a1**	**0.614**	**0.072**	**8.519**	**0.000**	**0.472**	**0.756**
	ENTR	a2	-0.123	0.082	-1.488	0.137	-0.288	0.037
	HS	a3	0.076	0.062	1.214	0.225	-0.044	0.200
	DEV	a4	0.055	0.064	0.849	0.396	-0.072	0.179
	GF	a5	0.079	0.069	1.140	0.254	-0.056	0.215
	EXP	a6	-0.059	0.057	-1.047	0.295	-0.166	0.057
	SSSE	a7	-0.060	0.060	-0.998	0.318	-0.177	0.059
Secure	Shame	b1	-0.114	0.068	-1.672	0.095	-0.249	0.019
Dismissive	Shame	b2	0.137	0.077	1.773	0.076	-0.020	0.281
Preoccupied	Shame	b3	0.111	0.065	1.701	0.089	-0.018	0.238
Fearful	Shame	**b4**	**0.179**	**0.063**	**2.833**	**0.005**	**0.060**	**0.310**

Indirect effects		Path	Parameter estimate	Standard error	z-value	p-value	95% CI Lower limit	95% CI Upper limit
CSE-Shame	Secure	a1b1	-0.070	0.042	-1.643	0.100	-0.157	0.011
CSE-Shame	Dismissive	a1b2	0.084	0.049	1.727	0.084	-0.012	0.177
CSE-Shame	Preoccupied	a1b3	0.068	0.041	1.644	0.100	-0.010	0.152
CSE-Shame	Fearful	**a1b4**	**0.110**	**0.041**	**2.667**	**0.008**	**0.035**	**0.199**
ENTR-Shame	Secure	a2b1	0.014	0.014	0.992	0.321	-0.005	0.048
ENTR-Shame	Dismissive	a2b2	-0.017	0.016	-1.036	0.300	-0.055	0.007

ENTR-Shame	Preoccupied	a2b3	-0.014	0.013	-1.028	0.304	-0.045	0.005
ENTR-Shame	Fearful	a2b4	-0.022	0.018	-1.239	0.215	-0.064	0.006
HS-Shame	Secure	a3b1	-0.009	0.009	-0.917	0.359	-0.030	0.007
HS-Shame	Dismissive	a3b2	0.010	0.011	0.923	0.356	-0.007	0.037
HS-Shame	Preoccupied	a3b3	0.008	0.009	0.886	0.376	-0.005	0.032
HS-Shame	Fearful	a3b4	0.014	0.013	1.048	0.295	-0.007	0.044
DEV-Shame	Secure	a4b1	-0.006	0.010	-0.652	0.514	-0.029	0.008
DEV-Shame	Dismissive	a4b2	0.007	0.011	0.692	0.489	-0.011	0.033
DEV-Shame	Preoccupied	a4b3	0.006	0.009	0.690	0.490	-0.009	0.026
DEV-Shame	Fearful	a4b4	0.010	0.013	0.760	0.447	-0.013	0.040
GF-Shame	Secure	a5b1	-0.009	0.011	-0.849	0.396	-0.034	0.008
GF-Shame	Dismissive	a5b2	0.011	0.013	0.842	0.400	-0.009	0.042
GF-Shame	Preoccupied	a5b3	0.009	0.011	0.829	0.407	-0.006	0.035
GF-Shame	Fearful	a5b4	0.014	0.014	0.990	0.322	-0.010	0.046
EXP-Shame	Secure	a6b1	0.007	0.008	0.850	0.395	-0.009	0.023
EXP-Shame	Dismissive	a6b2	-0.008	0.010	-0.820	0.412	-0.031	0.009
EXP-Shame	Preoccupied	a6b3	-0.007	0.008	-0.779	0.436	-0.027	0.006
EXP-Shame	Fearful	a6b4	-0.011	0.011	-0.935	0.350	-0.035	0.010
SSSE-Shame	Secure	a7b1	0.007	0.009	0.765	0.444	-0.007	0.028
SSSE-Shame	Dismissive	a7b2	-0.008	0.010	-0.788	0.431	-0.033	0.009
SSSE-Shame	Preoccupied	a7b3	-0.007	0.009	-0.778	0.436	-0.028	0.007
SSSE-Shame	Fearful	a7b4	-0.011	0.012	-0.891	0.373	-0.038	0.011
Total effects		Path	Parameter estimate	Standard error	z-value	p-value	95% CI Lower limit	95% CI Upper limit
CSE-Shame	Secure	**c1+a1b1**	**-0.313**	**0.079**	**-3.980**	**0.000**	**-0.467**	**-0.161**
CSE-Shame	Dismissive	**c8+a1b2**	**-0.437**	**0.075**	**-5.839**	**0.000**	**-0.581**	**-0.285**
CSE-Shame	Preoccupied	**c15+a1b3**	**0.371**	**0.079**	**4.692**	**0.000**	**0.217**	**0.529**
CSE-Shame	Fearful	**c22+a1b4**	**0.229**	**0.073**	**3.113**	**0.002**	**0.085**	**0.371**

ENTR-Shame	Secure	c2+a2b1	0.071	0.083	0.861	0.389	-0.085	0.238
ENTR-Shame	Dismissive	c9+a2b2	0.127	0.082	1.542	0.123	-0.038	0.287
ENTR-Shame	Preoccupied	c16+a2b3	0.004	0.088	0.050	0.960	-0.179	0.176
ENTR-Shame	Fearful	**c23+a2b4**	**-0.162**	**0.081**	**-1.992**	**0.046**	**-0.324**	**-0.002**
HS-Shame	Secure	**c3+a3b1**	**-0.277**	**0.066**	**-4.170**	**0.000**	**-0.405**	**-0.143**
HS-Shame	Dismissive	**c10+a3b2**	**0.272**	**0.062**	**4.372**	**0.000**	**0.144**	**0.388**
HS-Shame	Preoccupied	c17+a3b3	-0.107	0.064	-1.659	0.097	-0.231	0.019
HS-Shame	Fearful	**c24+a3b4**	**0.369**	**0.053**	**6.905**	**0.000**	**0.264**	**0.473**
DEV-Shame	Secure	c4+a4b1	-0.144	0.075	-1.917	0.055	-0.292	0.005
DEV-Shame	Dismissive	c11+a4b2	0.094	0.072	1.306	0.192	-0.047	0.234
DEV-Shame	Preoccupied	c18+a4b3	0.061	0.081	0.758	0.448	-0.095	0.219
DEV-Shame	Fearful	**c25+a4b4**	**0.153**	**0.073**	**2.106**	**0.035**	**0.010**	**0.295**
GF-Shame	Secure	c5+a5b1	0.006	0.064	0.092	0.927	-0.118	0.132
GF-Shame	Dismissive	c12+a5b2	0.102	0.065	1.561	0.118	-0.028	0.226
GF-Shame	Preoccupied	c19+a5b3	0.036	0.072	0.500	0.617	-0.107	0.174
GF-Shame	Fearful	c26+a5b4	0.030	0.064	0.465	0.642	-0.092	0.156
EXP-Shame	Secure	c6+a6b1	0.067	0.057	1.177	0.239	-0.044	0.178
EXP-Shame	Dismissive	c13+a6b2	0.034	0.063	0.543	0.587	-0.088	0.156
EXP-Shame	Preoccupied	c20+a6b3	0.009	0.060	0.149	0.882	-0.105	0.133
EXP-Shame	Fearful	c27+a6b4	-0.029	0.057	-0.519	0.604	-0.138	0.083
SSSE-Shame	Secure	**c7+a7b1**	**0.244**	**0.067**	**3.620**	**0.000**	**0.113**	**0.376**
SSSE-Shame	Dismissive	c14+a7b2	-0.050	0.070	-0.720	0.471	-0.190	0.088
SSSE-Shame	Preoccupied	c21+a7b3	0.046	0.073	0.624	0.533	-0.094	0.191
SSSE-Shame	Fearful	**c28+a7b4**	**-0.154**	**0.065**	**-2.359**	**0.018**	**-0.284**	**-0.028**

Note: Standardised variables are used in analyses therefore the parameter estimates are standardised parameters. Significant statistics are highlighted in bold. Covariances have been omitted from this table but can be found via Open Science Framework (see Declarations for link). CSE = Contingent self-esteem, ENTR = Entitlement Rage, HS = Hiding the self, DEV = Devaluing, GF = Grandiose Fantasies, EXP = Exploitativeness, SSSE = self-sacrificing self-enhancement.
